# DeepFeature: feature selection in nonimage data using convolutional neural network

**DOI:** 10.1093/bib/bbab297

**Published:** 2021-08-06

**Authors:** Alok Sharma, Artem Lysenko, Keith A Boroevich, Edwin Vans, Tatsuhiko Tsunoda

**Affiliations:** Laboratory for Medical Science Mathematics, RIKEN Center for Integrative Medical Sciences, Yokohama 230-0045, Japan; Laboratory for Medical Science Mathematics, RIKEN Center for Integrative Medical Sciences, Yokohama 230-0045, Japan; Laboratory for Medical Science Mathematics, RIKEN Center for Integrative Medical Sciences, Yokohama 230-0045, Japan; STEMP, University of the South Pacific, Suva, Fiji; Laboratory for Medical Science Mathematics, Department of Biological Sciences, Graduate School of Science, The University of Tokyo, Tokyo 113-0033, Japan

**Keywords:** Feature selection, Non-image data, Convolutional neural network, Omics data, DeepInsight

## Abstract

Artificial intelligence methods offer exciting new capabilities for the discovery of biological mechanisms from raw data because they are able to detect vastly more complex patterns of association that cannot be captured by classical statistical tests. Among these methods, deep neural networks are currently among the most advanced approaches and, in particular, convolutional neural networks (CNNs) have been shown to perform excellently for a variety of difficult tasks. Despite that applications of this type of networks to high-dimensional omics data and, most importantly, meaningful interpretation of the results returned from such models in a biomedical context remains an open problem. Here we present, an approach applying a CNN to nonimage data for feature selection. Our pipeline, DeepFeature, can both successfully transform omics data into a form that is optimal for fitting a CNN model and can also return sets of the most important genes used internally for computing predictions. Within the framework, the Snowfall compression algorithm is introduced to enable more elements in the fixed pixel framework, and region accumulation and element decoder is developed to find elements or genes from the class activation maps. In comparative tests for cancer type prediction task, DeepFeature simultaneously achieved superior predictive performance and better ability to discover key pathways and biological processes meaningful for this context. Capabilities offered by the proposed framework can enable the effective use of powerful deep learning methods to facilitate the discovery of causal mechanisms in high-dimensional biomedical data.

## Introduction

Gene set selection facilitates interpretation of high-dimensional omics data by reducing it to components of greatest relevance and then allowing them to be linked to specific functional themes (e.g. using methods like process and pathway enrichment analysis) and to extract meaningful clues for clarifying biological mechanisms. Other important applications include disease diagnostics, where the goal is to produce gene sets small enough to be used for diagnostic tests in clinical practice.

Traditional machine learning (ML) algorithms (such as support vector machines [1], random forest, RF [2] and logistic regression [3]) are the ones most commonly applied in classification and feature selection (or gene selection) of nonimage data. Primarily, a $d\times 1$ column vector is supplied to an ML algorithm to find a smaller set of features and/or to classify samples into one of defined classes. In the medical field, ever increasing data complexity is pushing the limits of ML algorithms to extract relevant information for phenotype identification related to disease diagnosis and analysis. In this respect, the selection of a small subset of critical elements or genes from a larger set has become a critical step. The element or gene selection (also known as feature selection) problem is not limited to genomic data analysis, but is an important process in many areas of research. The reliability of ML algorithms to find a subset of genes is mostly determined by the feature selection, feature extraction and classification steps.

On the other hand, convolutional neural networks (CNNs) are a class of deep learning architectures that have shown promising results and gained widespread attention in all types image analysis [4–14]. CNN takes an input image (a p × q feature matrix) and through its hidden layers conducts feature extraction and classification (note for 1-dimensional (D) CNN the input is not of $\mathrm{p}\times \mathrm{q}$ size as the applied input is not an image sample). The 2-dimensional CNN is generally referred to as a CNN and this paper will also follow suit. One of the key advantages of CNNs are their high efficiency, i.e. fewer samples and less training time are needed to achieve good levels of performance. This led to their high popularity in a myriad of cutting-edge commercial applications (e.g. driverless cars). A CNN has several advantages: it automatically derives important features from spatially coherent pixels, it finds higher-order image statistics and nonlinear correlations, it requires less neurons as its convolution architecture can processes data for its receptive fields (or small subareas), allowing a deeper network with fewer parameters and its receptive fields share the coefficients and biases, reducing the memory footprint [15]. In a local region, an image is comprised of spatially coherent pixels; i.e. similar information is shared by the pixels near each other. CNNs take into account the neighborhood information by extracting features from the adjacent pixels. On the other hand, ML techniques discard the neighborhood information and assume every element of a sample to be independent. Therefore, to get the maximum performance from a CNN, the adjacent pixels of a 2D input feature matrix should have reasonable coherence. Fortunately, for CNNs, the images utilized are usually a representation of physical entities and, therefore, do not require pixel rearrangement, as the lenses of camera correctly pass the appropriate light shades of animate or inanimate objects to the pixels. Previous attempts to apply CNN to nonimage data were restricted to 1D CNN architectures [16–18]. The input to 1D CNNs are in the form of feature vectors and therefore they cannot deal with images.

One possible way CNNs can process nonimage samples is by first converting it to an image sample considering spatially coherent pixels in its local regions, i.e. the pixels close to each other share similar information sometimes with patterns. The arbitrary arrangement of pixel locations can induce an unfavorable impact on the feature extraction and classification performance of CNN architecture. Therefore, the order of neighboring pixels in an image utilized by CNN is no longer independent as they were in ML techniques [19]. The DeepInsight approach [19] pioneered a variant of this strategy that used t-SNE [20] for element arrangement, followed by mappings, feature extraction and classification steps. The element arrangement is done by positioning elements or genes within a 2D pixel frame based on their relative similarities, followed by the mapping of element values onto these locations. This approach ubiquitously transforms nonimage samples into images suitable for CNNs. To our knowledge, it was the first approach to convert various kinds of nonimage data to image forms for the application of CNN architecture. Buturivić and Miljković [21] introduced a variant of DeepInsight approach where tabular data (generally a nonimage data in the arrangement having rows and columns) for CNN (with ResNet architecture) is used by transforming rows of tabular data as an image filter, and then by applying it to a fixed-base image. They applied their approach to gene expression data derived from blood samples of patients with bacterial or viral infections and showed that this pipeline can outperform many ML algorithms. Kanber [22] applied the DeepInsight approach to the sparse data of the MINST database of 70k samples and showed it had superior performance than a state-of-the-art ML (RF) method. DeepInsight approach has been applied in various other projects [23–42].

The usability of DeepInsight based model has also been noticed in data science online platform (such as Kaggle.com). Recently, a competition was organized by the Connectivity Map, a project within the Broad Institute of MIT and Harvard, the Laboratory for Innovation Science at Harvard (LISH) and the NIH Common Funds Library of Integrated Network-Based Cellular Signatures (LINCS), on the Kaggle.com platform (https://www.kaggle.com/c/lish-moa/overview). The title of the competition was Mechanisms of Action (MoA) predictions. The organizers posed a problem where it was required to develop an algorithm that can classify drugs based on their biological activities. A total of 4373 teams participated and submitted their models. The winning team of Peng *et al.* [43] applied DeepInsight feature mapping with EfficientNet-B3 NS model and ResNeSt model with five other models to score rank 1 out of the total of 4373 teams (https://www.kaggle.com/c/lish-moa/discussion/201510). The implementation and description of DeepInsight part by Peng *et al.* [43] can be accessed at https://www.kaggle.com/c/lish-moa/discussion/195378.

To date there are very few studies about how to perform feature selection by CNN for nonimage samples, such as finding a subset of genes. In this work, we focus on developing a methodology to show that gene selection can be done using CNNs. An obvious comparable method for this type of analysis is differential gene expression analysis (DGE) and therefore it is important to highlight some key differences between it and the proposed DeepFeature method. In most typical types of DEG analysis genes are processed individually and the comparisons are made between two or more conditions based on some variation of a linear model. The analysis would return all the genes found to be substantially different, though ability to meet the selection threshold is heavily influenced by the variance and magnitude of the expression, which may not necessarily align with the overall importance of these genes to the condition of interest. In contrast, deep learning classifier at the core of the DeepFeature model performs selection by considering all of the available genes simultaneously (which allows collinearity to be exploited to compensate for noise) and the priority is solely given to the overall predictive importance of the gene within the context of the selected set. Due to these differences in selection criteria DeepFeature can offer a rich, complementary perspective to that of the traditional methods. As illustrated by the analysis reported in this paper, DeepFeature-selected gene sets are both very different from more traditional approaches like lasso and analysis of variance (ANOVA), but also appeared to be better aligned with meaningful biological mechanisms and therefore consistently achieved higher enrichment for key pathways and functional groups.

**Figure 1:**
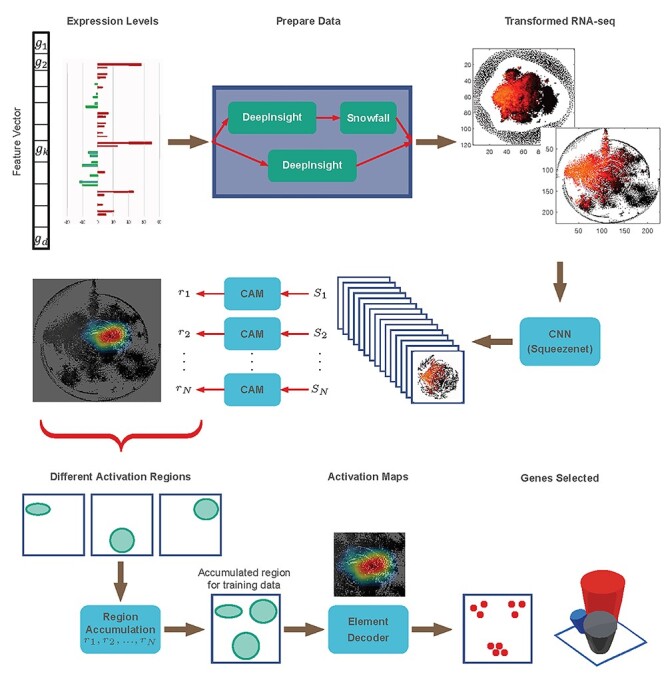
An overall DeepFeature procedure for feature selection using CNN.

The hidden layers of CNNs can reveal complex mechanisms (such as pathways) for nonimage samples. The development of CNNs is inspired by biological processes to perform feature extraction from image patterns [14, 44, 45]. Both in industries (e.g. as driverless cars) and academia, the usage of CNN is becoming increasingly important. It has been primarily used for image processing but now CNNs are expanded to many fields. Numerous research can be cited showing CNNs reveal a complex pattern in the data to achieve superior performance [14, 46, 47]. It is possible that the proper utilization of CNN with DeepInsight feature mapping can also reveal complex mechanisms for nonimage samples. The same methodology can be extended to other kinds of nonimage cases and is not restricted to genomic or transcriptomic data. To this end, the proposed Feature Selection algorithm via CNNs for nonimage samples, abbreviated as DeepFeature, was developed (Figure 1). The DeepFeature approach encompasses four main steps: element arrangement, feature selection, feature extraction and classification (see Supplementary File 1 for the definition about these terms). The steps of DeepFeature are discussed in the Materials and methods section.

The innovation and/or contributions of this paper are as follows. DeepFeature pipeline is introduced where feature selection can be performed for nonimage samples (or tabular data) via CNNs. Snowfall compression algorithm is developed to allow more elements of data in a fixed pixel frame. Region accumulation and element decoder (READ) are introduced to find genes or elements from the activation maps. In addition to delivering high-classification performance, DeepFeature also offers a powerful means for the identification of biologically relevant gene sets. When applied to the task of cancer classification, our DeepFeature approach was able to identify coherent sets with significant enrichment of genes in cancer-associated pathways from MSigDB and a gold-standard reference set. Further analysis of these results suggested biologically meaningful connections of potential interest to our understanding of the differences between major cancer types.

## Results

The results are produced by following an overall procedure of DeepFeature (Figure 1). The model takes nonimage data, e.g. transcriptomic or RNA-seq data, and finds a subset of genes or elements via CNN. The model carries the following steps: image transformation by DeepInsight, optional Snowfall compression to accommodate more elements in a pixel-frame, classification via SqueezeNet model of CNN architecture [48], identification of activation maps using CAM [49], discovery of the overall activated regions for a category or class via the region accumulation step and the decoding of gene subsets by the element decoder procedure.

First, we compared the number of selected genes and classification performance for the TCGA cancer study identification task using different ML algorithms (Table 1). Lasso gave 1018 nonzero coefficients and as a result it discarded all other elements or genes. To perform enrichment analysis, we limited the size of gene subset to be around 1000–2000. In this respect, for ANOVA and variable genes method, 1000 genes were extracted. These techniques did not provide class specific gene subsets, however, gave one subset of genes for the all 10 cancer studies. The classification accuracy was computed on gene subsets using the RF classifier.

On the other hand, the logistic regression+RF method generates models for each cancer study $i$ (for *i* = 1,2, … 10) with coefficients ${w}_j^i$, where $j=1,2,\dots, d$ and $d$ depicts the number of features or genes. If we take the average coefficients over 10 classes and find top $r$ features, we can then get one subset of genes for all the 10 cancer studies. However, it will not be as useful because in that case it will not be possible to obtain class specific features. Considering each model separately, we take an absolute value or modulus of the coefficients and arrange them in descending order to find specific features related to each cancer study (details are discussed in the Materials and methods section).

Therefore, these algorithms, with the exception of logistic regression+RF, provide one subset of genes for all the categories. However, in some cases pairwise analysis is possible (e.g. by using post-hoc Tuckey’s test [50]). In such a case, a total of ${m}\choose{2}$ subsets are to be generated, where $m$ is the number of categories. On the other hand, logistic regression+RF provides a separate model for each cancer study, and with some modifications, it is possible to obtain class dependent features. This way, it is possible to find a subset of genes corresponding to a particular cancer class. Other methods, such as Seurat [51], pcaReduce [52], TSCAN [53] and SINCERA [54], also produce gene subsets. To make sure the evaluation is meaningful, DeepFeature method was only compared with approaches that are also capable of returning individual sets of features for each class.

For DeepFeature, three different visualization tools were used/developed to plot feature locations on a 2D-plane. These methods were (1) t-SNE, (2) t-SNE with Snowfall and (3) PHATE [55] (see Supplementary File 1 for details).

DeepFeature achieved 97–98% classification accuracy on the independent test set when using t-SNE (with or without Snowfall algorithm), and 96.8% accuracy when using PHATE.

For DeepFeature with t-SNE and Snowfall, four distances (Chebychev, correlation, cosine and Hamming) were used which gave a subset of 5228 genes (see Supplementary Tables S1.1–S1.3 for hyperparameter details, Supplementary Figure S1.2 for an illustration of corresponding activations and Supplementary Figure S1.3 for gene subsets per cancer study). This subset of genes was further processed with DeepFeature using Hamming distance which gave 1806 genes. The details about DeepFeature execution and results at various stages that eventually led to the selection of 1806 genes can be found in SupplementaryFile 1.

The same four distances and additional iteration were also used in the case of DeepFeature with t-SNE (no Snowfall). The two resulting subsets were of 1914 genes and 962 genes (see Supplementary Table S1.4, Supplementary Figures S1.4 and S1.5 in Supplementary File 1 for details). Two different sizes of gene subsets were generated from a particular model to examine whether gene subset size effects pathway enrichment.

Lastly, the PHATE algorithm was used for visualization with DeepFeature, which gave 1569 genes (see Supplementary Table S1.5, Supplementary Figures S1.6 and S1.7 in Supplementary File 1 for details).

Different visualization algorithms (t-SNE with or without Snowfall and PHATE) were applied in DeepFeature and the resulting gene subsets were examined for enrichment of genes in pathways of interest.

**Figure 2:**
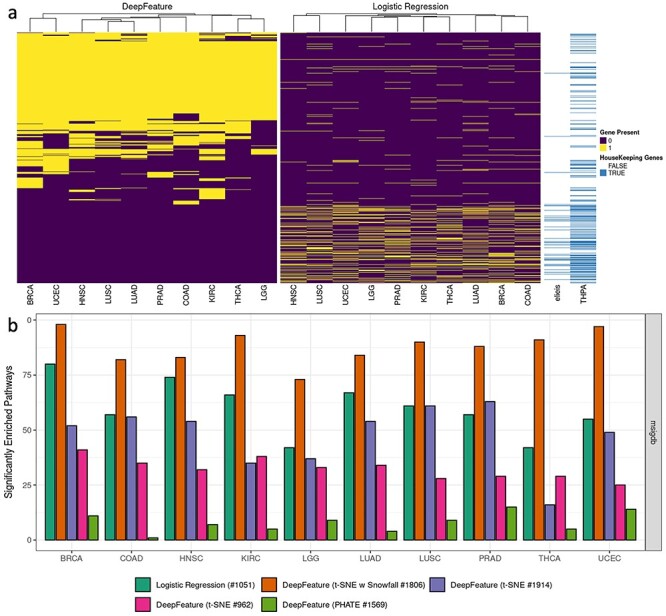
(**A**) Commonality of gene selected by DeepFeature (t-SNE + Snowfall) and logistic regression techniques. Each row represents a gene and yellow denotes that gene was selected for that cancer study (column). Housekeeping genes, as identified by two annotation sources, are shown in blue. (**B**) Enrichment analysis in MSigDB C6 gene sets. The number of significantly enriched cancer sets for genes selected by DeepFeature (orange, pink, purple and light green) and logistic regression (dark green) for each of the 10 cancer studies. DeepFeature (orange) produced higher counts than logistic regression for MSigDB for all the cancer studies.

DeepFeature is also capable of finding different gene subsets belonging to different cancer studies. As discussed above, applying different visualization tools resulted in different gene subsets of sizes 962, 1569, 1806 and 1914 genes for each of the 10 cancer studies. We applied enrichment analysis on these 962, 1569, 1806 and 1914 gene subsets. DeepFeature using t-SNE + Snowfall gave a 1806 gene subset and the number of genes for each cancer study ranged between 930 and 1450 (see Supplementary Figure S2.1 in Supplementary File 2). DeepFeature using t-SNE gave the 962 and 1914 gene subsets. In the case of 962 gene set, between 307 and 679 genes per cancer study were detected (Supplementary Figure S2.1 in Supplementary File 2). For the 1914 gene set, between 405 and 1571 genes per cancer subtype were found. DeepFeature using PHATE gave 1569 genes, and in this case between 24 and 621 genes per cancer study were found (see Supplementary Figure S2.1). DeepFeature using t-SNE + Snowfall more consistently identified a common set of genes selected for all cancer studies (Figure 2A and Supplementary Figure S2.2 for performance evaluation of all the algorithms; and, Supplementary Figure S2.3 for overlap of gene subsets among all the methods including gene annotation). DeepFeature using t-SNE (962 and 1914 genes) performed satisfactorily, however, it did not outperform DeepFeature with t-SNE + Snowfall (Figure 2B). This shows that using Snowfall algorithm, the enrichment is improved while using DeepFeature. The PHATE visualization method for DeepFeature did not perform well and consistently showed inferior performance (Figure 2B). The number of selected genes for PHATE (1569) was also higher than t-SNE (962), but the number of significantly enriched pathways was less than half for most of the cancer studies. Interestingly, in addition to this overall trend, logistic regression and DeepFeature appear to preferentially select the nonoverlapping sets of genes relative to each other. Still, DeepFeature was far better in recovering significantly enriched cancer-relevant pathways (Figure 2B). This suggests both that the proposed algorithm is better at discovering biologically coherent groupings of genes and that most of these grouping also appear to be highly relevant to cancer-associated processes. Notably, sets of genes from DeepFeature are also depleted for housekeeping genes (Figure 2A). As housekeeping genes usually tend to have stable expression across all cell types and tissues, this result is likely an indication that fewer false positive genes, which are unlikely to be of interest, were chosen.

**Table 1:** Classification accuracy and gene selection using ML and DeepFeature algorithms

Machine learning methods	#Genes selected	Classification accuracy
ANOVA+RF	1000	94.2%
Lasso+RF	1018	96.0%
Variable genes+RF	1000	92.8%
Logistic regression+RF	1051	96.6%
DeepFeature (t-SNE)	962	97.6%
DeepFeature (t-SNE)	1914	96.6%
DeepFeature (t-SNE with SnowFall)	1806	97.9%
DeepFeature (PHATE)	1569	96.8%

To compare these results with previously proposed gene sets suitable for identification of cancer types, an additional comparison was made with a ‘hallmark gene sets’ and ‘chemical and genetic perturbations’ collections from the MSigDB database (Supplementary Figure S2.4) that aim to characterize coherently expressed biological processes and signatures for key biological and clinical sets, respectively. Although the overlap with this dataset was somewhat marginal, the topmost enriched pathways recovered from this set were still highly relevant. From the hallmarks collection, most significant gene set was ‘Epithelial mesenchyma transition’, which is a core process in both tissue differentiation and initiation of cancer metastasis. Other cell proliferation and differentiation processes near the top of the list were angiogenesis (3rd highest) and myogeneis (6th); notably the former of these encompasses genes responsible for regulation of new blood vessel formation, which is crucial for tumor growth and cancer progression. Core cancer signaling pathways mediated by KRAS, TNF-alpha and JAK/STAT proteins were also found near the very top of the list. Other recovered mechanisms commonly associated with cancer were coagulation (2nd highest, typically abnormally enhanced in cancer patients) inflammatory response (7th, associated with anti-tumor immune response) and hypoxia (5th, a common condition that occurs in regions of tumor that grow too rapidly to ensure adequate oxygen supply). In summary, this analysis has confirmed that DeepFeature method was able to consistently identify important processes well-known to be highly relevant for cancer and therefore may be also be potentially promising for the discovery of new candidate pathways and genes.

To explore possible underlying biological meaning behind these selected gene groupings, each set of cancer study-specific genes was evaluated for enrichment of specific pathways in Reactome database (Supplementary File 3). This analysis revealed that DeepFeature consistently identified genes belonging to the ‘Extracellular matrix organization’ pathway (Reactome: R-HSA-1474244), which was the top group for all cancer studies—both by number of genes and enrichment significance (see Supplementary File 3 for top pathways). The importance of this pathway is in line with current understanding of its wider biological role, where it is known to be both highly diverse in its expression across different tissues and subject to dysregulation in cancer [56]. Another two pathways that were highly enriched across all cancer studies were ‘Signaling by Receptor Tyrosine Kinases’ (R-HSA-9006934) and ‘GPCR ligand binding’ (R-HSA-500792). Tyrosine kinases mediate both cell proliferation and apoptosis, and their importance for multiple types of cancers is relatively well-studied [57]. Interestingly, although it has been observed that different GPCRs are expressed in different cancers and some of them may be suitable for use as biomarkers [58], overall roles of specific genes in cancer is not well understood and the ligands of many GPCRs still have not been identified [59]. Further analysis of these DeepFeature results, like holistic analysis of which parts of specific pathways were found to be important for different types, may help to explain key cancer study specific differences, and allow to connect the GPCR signaling to other better understood mechanisms of oncogenesis.

## Discussion

The performance evaluation of DeepFeature is gauged by both finding significantly enriched pathways within the selected subset of genes and classification accuracy on the independent test set. As expected, promising results were obtained when compared with the state-of-the-art ML techniques. The gene enrichment results for DeepFeature were reasonably sound compared with the benchmarked ML methods. The classification accuracy on the independent test set was ~98%, which is better than the ML technique. This shows that deep learning architectures have a possibility to provide solutions for biomarker discovery, genomic analysis for a variety of input samples ranging from RNA-seq to various omics data. In general, this method is suitable for applications where given data is in a nonimage form.

The three visualization methods used in the DeepFeature pipeline were t-SNE, PHATE and t-SNE + Snowfall. Though the Snowfall algorithm adds distortion in the visualization of t-SNE, t-SNE + Snowfall performed better than t-SNE as measured by gene enrichment analysis. The distortion is introduced when the image is transformed from a Cartesian coordinate system to the fixed size pixel framework, as many features overlap, i.e. various features having the same pixel location. This overlapping reduces the chance of a feature to be selected. In such a case, the application of Snowfall is to find nearby pixel locations for features and expose more features to selection. If we could have very large pixel frame size where no overlaps occur while transforming from Cartesian coordinate system to pixel frame, then perhaps the application of Snowfall will not be useful as distortion is added by it while adjusting the features in the nearby pixel locations. Nonetheless, in a given fixed size pixel framework, the application of Snowfall for DeepFeature was found to be useful in finding more meaningful features or genes. On the other hand, although PHATE [55] has been promoted to be an advanced visualization technique, it did not perform well when compared with t-SNE in the DeepFeature pipeline. This inferior performance may be attributed to the error induced while converting from Cartesian coordinates system to pixel coordinates system as many elements are susceptible to overlap due to the fixed size of pixel frame.

The feature selection results indicated that DeepFeature was much better than alternative algorithms at recovering biologically meaningful groups of genes that were relevant to classes (phenotypes) of interest. A notable advantage of the method is its ability to narrow down the set of candidate genes even in cases where the differences are very substantial, like different cancer types. On the contrary, under such circumstances approaches like DGE return very large number of significant hits and therefore are not useful in sufficiently reducing the candidate list(s) to allow meaningful interpretation. Therefore, the algorithm is likely to have great utility for tasks like prioritization of diagnostical signatures and interpretation of complex multi-omics data.

It is now generally accepted that in order to fully understand the dynamic quintessential complexity of cancer, it is essential to profile and collect data using a wide array of possible methods. As a result, a typical study commonly needs to consider wide array of possible data types, possibly including images, clinical records, as well as protein-coding and noncoding RNA expression profiles and full genome sequencing. Understanding mechanisms and discovery of clinically relevant subtypes frequently require appropriate aligning of these different types of data and, most often, subsequent use of ML algorithms to identify meaningful patterns. Among current generation of approaches, deep learning-based methods offer the greatest flexibility and can potentially allow fuller automation and more comprehensive integration of these different types. In principle, by combining different specialized encoder layers, any types of data can be converted into a set of inter-compatible embeddings and used within the same ML system. This innate flexibility is difficult to match using other methods and is particularly valuable for mining complex multimodal datasets.

Good correspondence of identified gene sets with known gold standard cancer-associated pathways is particularly promising as a means of interpreting the deep learning results from a biological perspective. Despite many recent advances, deep learning still commonly has a reputation for generating high quality but ultimately ‘black box’ models, where discovering how an algorithm arrived at a particular conclusion is very challenging. However, given that structures of key cancer pathways are very well understood, their high degree of overlap with DeepFeature results could be crucial for correctly contextualizing the importance of individual genes and offer possible explanations for why they were selected by the algorithm.

## Materials and methods

We obtained RNA-seq data from the TCGA project. To maintain large enough categories, only 10 cancer studies were considered, namely, TCGA-BRCA, TCGA-COAD, TCGA-HNSC, TCGA-KIRC, TCGA-LGG, TCGA-LUAD, TCGA-LUSC, TCGA-PRAD, TCGA-THCA and TCGA-UCEC. A total of 6280 HTSeq-FPKM-UQ expression files were downloaded using the GDC data transfer tool.

From these, 64 files were removed for sharing submitter IDs, resulting in a final total of 6126 samples. The FPKM-UQ files contain expression for 60 483 genes. In this study, we only used the 19 086 genes classified as protein-coding genes by the HUGO Gene Nomenclature Committee (download date: 22 November 2019). Next, we will describe the DeepFeature model.

An overall procedure for feature selection using CNN (Figure 1). The model takes nonimage data, e.g. transcriptomic or RNA-seq data, and finds a subset of genes or elements via CNN. The transformation of nonimage samples to image samples is done following the element arrangement step of the DeepInsight model. A Snowfall compression algorithm is developed to fit more elements in a given pixel-frame to enable every possible gene to be part of the selection. Here three visualization methods are used: t-SNE, t-SNE with Snowfall and PHATE. Image samples obtained from the DeepInsight and Snowfall algorithms (if selected) are submitted to the CNN model (using SqueezeNet architecture). The feature extraction and classification are performed by CNN. Feature selection is performed collectively by the class activation maps (CAMs), region accumulation and element decoder (READ). CAM are used to find activations of each sample. The activations for individual samples are integrated to find active regions for a class or category at the region accumulation step. The accumulated regions (for one or all classes) define pixel locations of interest for categorization of samples. These selected pixels are decoded to provide a subset of elements at the element decoder step. If the number of selected genes is higher than the desired number of genes, then the whole procedure can be executed again with the selected genes as input to find further subsets of genes. Repeating these steps will reduce the number of selected genes. This way the feature selection is performed with DeepFeature method.

### DeepFeature: feature selection for nonimage data using CNN

This section defines the proposed DeepFeature methodology. The constituents of the model are (1) image transformation by DeepInsight, (2) Snowfall compression to enable more elements in a pixel-frame, (3) SqueezeNet model of CNN architecture, (4) CAM model to find activation maps, (5) region accumulation to obtain overall activated regions for a category or dataset and (6) element decoder to decode genes from active regions. These steps are discussed below.

### DeepInsight: nonimage to image conversion for CNN

DeepInsight transforms a nonimage sample to a well-organized image form by effectively arranging elements while considering neighborhood information. The feature extraction and classification tasks are done by CNN. DeepInsight integrates three steps: (1) element arrangement, (2) feature extraction and (3) classification. This approach of element arrangement can be useful in uncovering hidden mechanisms (e.g. pathways). In this way, the relative importance of features for assignment of samples to particular classes can be better understood. An input feature vector is transformed to a feature matrix using t-SNE [20], kernel PCA [60], PHATE [55] or UMAP [61], and then the smallest rectangle containing all the elements is found using the convex hull algorithm. A necessary rotation is performed to align the image, and then Cartesian coordinates are converted to pixel coordinates. After that, mapping of element values onto pixel locations is performed to construct an image of a feature vector. The details of image transformation procedure have been previously described in our earlier work [19].

### Snowfall compression algorithm

If the dimensionality of a sample with *d* elements, $x\in{\mathbb{R}}^d$, is very large, then it becomes very difficult to place all the elements in a given pixel frame of size $m\times n$. Therefore, the question is, how to compress, such that all the elements can be arranged in the same pixel size, while maintaining the data topology. There are two ways of performing compression, quantized compression and non-quantized compression. In quantized compression, two or more elements can overlap, i.e. these elements will have an identical pixel location. In this case, the values of elements are averaged at that particular pixel location. On the other hand, in non-quantized compression, no overlap occurs and each element maintains a unique pixel location and thus there is no averaging of their values at a given location. The Snowfall algorithm is a non-quantized compression algorithm. However, depending upon the memory requirements of a given hardware by CNN, the size of the pixel frame can be adjusted such that all the elements are represented in the frame, but with quantized compression. See Supplementary File 4 for details about the Snowfall compression algorithm.

It is important to note that when an image in transformed from Cartesian coordinates to pixel coordinates, it brings distortion due to the limited or fixed size of the pixel frame. Due to limited size, many features may overlap on the same location and it would become difficult to perform feature selection. The Snowfall algorithm tries to find nearby empty pixel locations of such features so that overlapping can be minimized. Therefore, it tries to make features visible to CNN by relocating them to neighboring points thereby helping to perform feature selection.

### CNN architecture for feature selection and classification

Since class activation maps (CAMs) [49] cannot be used for networks that have multiple fully connected layers at the output layer, we used SqueezeNet in this work [48]. Other series nets (e.g. AlexNet, VGG-16 and VGG19) can also be used to find CAMs.

**Figure 3:**
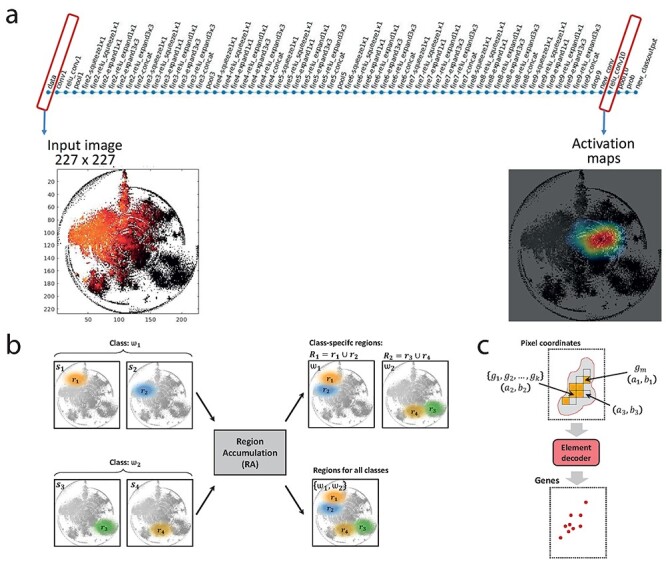
(**A**) SqueezeNet structure with 227 × 227 input image sample from DeepInsight and Snowfall algorithm, and corresponding class activation map at the output ReLu layer. (**B**) Region accumulation (RA) step for the DeepFeature method. (**C**) Element decoder for the DeepFeature method, the region R is supplied to the element decoder. A pixel under R could have three possibilities: a pixel representing only one gene g_m at location (a_1,b_1), a pixel representing multiple genes (g_1,g_2,…,g_k) at location (a_2,b_2), and a pixel that has no element, e.g. at location (a_3,b_3) (here a pixel value or Base is 1, see Supplementary File 1 for further discussion).

The SqueezeNet architecture of DeepFeature has fixed input size (see Figure 3A). The input image of size $N\times M$ is adjusted to $227\times 227$ due to the input image size requirement of 227 × 227 the first convolutional layer of SqueezeNet (*conv1*). Therefore, the input image size would be $227\times 227$. The activation maps are retrieved from the last connected ReLu layer (*ReLu_conv10*). After training the CNN model on the optimal hyperparameters, each new sample could be categorized into one of the classes or phenotypes (*new_classoutput*) at the output layer. Here, an RNA-seq gene expression sample was first converted to an image by DeepInsight and Snowfall compression algorithm, and then used as an input. At the input, the RNAseq sample can be visualized and a particular region leading to its identification can be analyzed at ReLu layer. The activation map at ReLu layer defines which localities of an image are of interest for decision making process. This activation map has three colors in order of preference as red, yellow and blue. The red zone is the most active and blue is the least (Figure 3A).

CNN has various hyperparameters such as momentum, L2 regularization and learning rate. These hyperparameters are tuned on the training set, and the model’s fitness is evaluated on the validation set by employing a Bayesian optimization technique for all the trials. The hyperparameters are selected to minimize the validation error. The test set is never been used in the training or model fitting steps. The chosen CNN hyperparameters produce the optimum performance on the validation set. Description of the parameters is further discussed in Supplementary File 1.

### Class activation maps (CAMs)

Zhou *et al.* [49] proposed CAMs which is an interesting addition of CNNs with global average pooling. CAMs reveals discriminatory image regions of a particular class or category of CNN used for classification. Here, the predicted scores of a category are mapped back to the previous convolutional layer to generate CAMs [49]. The region of image used for classification by CNNs can be visually observed by CAMs. The CAMs are fitted in the last convolutional layer at spatial location to perform global average pooling. The CAM for class $c$ is defined as

(1) \begin{equation*} {M}_{c\left(x,y\right)}={\sum}_k{\omega}_k^c{f}_{k\left(x,y\right)} \end{equation*}

where ${f}_k\big(x,y\big)$ depicts the activation of unit $k$ in the last convolutional layer at spatial location $\big(x,y\big)$, ${\omega}_k^c$ is the weight corresponding to class $c$ for unit $k$. The score for class $c$ can be obtained by ${S}_c={\sum}_{x,y}{M}_c\big(x,y\big)$.

### Region accumulation

The region accumulation (RA) step integrates regions of importance. Let a training set with $N$ samples be defined as $S=\big\{{s}_1,{s}_2,\dots, {s}_N\big\}$. Let the activation region corresponding to $N$ samples be given as $\mathcal{H}=\big\{{r}_1,{r}_2,\dots, {r}_N\big\}$; i.e. cardinality of $\mathcal{H}$ is same as $S$; i.e. $\Big|\mathcal{H}\Big|=\mid S\mid$. Let $c$ be the number of categories (or phenotypes) defined as $\Omega =\big\{{\omega}_1,{\omega}_2,\dots, {\omega}_c\big\}$. Each of the sample will have one of these categories; i.e. ${s}_i\in \Omega$ and ${r}_i\in \Omega$, for $i=1,2,\dots, N$. The overall region of the training data $S$ can be evaluated by performing *union operation* of individual regions; i.e. $R={r}_1\cup{r}_2\dots \cup{r}_N$ is the integrated region for all samples or for all categories. The region per class is also important to find genes belonging to a particular phenotype. In this case, ${R}_j={\cup}_{k=1}^{n_j}{\hat{r}}_k$, where ${n}_j$ is the number of samples in the subset represented by ${\omega}_j$ category, the region ${\hat{r}}_k\in \mathcal{H}$ for $k=1,2,\dots, {n}_j$ and belongs to a particular class, ${\hat{r}}_k\in{\omega}_j$. Basically, ${R}_j$ is the union of all regions depicting a particular class or phenotype. The active region size can vary depending on the threshold value. An illustration is given in Figure 3B where two samples $\big\{{s}_1,{s}_2\big\}$ are from category ${\omega}_1$, and another two samples $\big\{{s}_3,{s}_4\big\}$ are from ${\omega}_2$. The active region in each of the samples is depicted as $\big\{{r}_1,{r}_2,{r}_3,{r}_4\big\}$. All the four samples are processed via RA step, and it gives two outputs. In the first output, integrated regions ${R}_j$ (where $j=1,2$), belongs to individual categories are shown, i.e. active regions related to a particular phenotype. In the second output, all the active regions are integrated as $R$, depicting the necessary active zones for classification of two phenotypes.

### Element decoder

The output of RA is processed to the element decoder model. The pixels underneath the selected region are considered for this task. Figure 3C shows the element decoder system. In general, a pixel ${p}_i$ will have a normalized value $0\le{v}_i,\le 1$. The decoder will find the argument or index of this pixel ${p}_i$ located at $\big({a}_i,{b}_i\big)$, i.e. the unique elements or genes, ${G}_i$, that it contains. If the compression (see the section describing the Snowfall compression algorithm) is quantized then chances are high to get $\big|{G}_i\big|>1$, and if non-quantized then $\big|{G}_i\big|=1$, where $\mid \bullet \mid$ is its cardinality. In general, for some pixels $\big|{G}_j\big|=1$ and for some $\big|{G}_k\big|>1$ in a given pixel frame (where $j$ and $k$ are any two pixels).

For a region $R$, a subset of elements or genes will be obtained. It is possible to find a subset of genes for a particular class and also for all the classes. Furthermore, the element decoder can also reveal a subset of genes for a sample. It should be noted that the model can give different subsets of elements for different categories enabling class dependent findings.

### Reduction of elements through iteration

For genomic or transcriptomic data, the number of genes is normally very high and it becomes very difficult to fit all genes into a finite image size due to fixed hardware limitations. In this case, it is inevitable to get quantized images, i.e. some image pixels will carry multiple genes in a location. One might wonder, how to perform the selection on those batch genes (where batch gene refers to a set of two or more genes having the same pixel location in the frame). One may also want to reduce the number of selected elements. These issues can be addressed by running DeepFeature iteratively. The first iteration will find a subset of elements, which can be used as the input for the subsequent iteration. Continuing this procedure will reduce the number of elements.

### Logistic regression

Here we developed L2-regularized logistic regression model, i.e. each of the 10 cancer studies has its model as shown below [62].

(2) \begin{equation*} \underset{\mathbf{w}}{\min}\frac{1}{2}{\mathbf{w}}^T\mathbf{w}+C{\sum}_{j=1}^d\log \left(1+{e}^{-{y}_j{\mathbf{w}}^T{x}_j}\right), \end{equation*}

where $\frac{1}{2}{\mathbf{w}}^T\mathbf{w}$ is the L2 regularization term (Ridge), $i=1,2,\dots, 10$ and $d$ is the number of genes. We create models for all the 10 cancer studies and therefore get coefficients ${w}_j^i$ (where $j=1,2,\dots d$). Arranging the absolute of coefficients in the descending order, we get

(3) \begin{equation*} \left|{w}_1^i\right|>\left|{w}_2^i\right|>\dots >\mid{w}_m^i\mid \end{equation*}

This gives the top $m$ genes ${x}_j$. Since $i=1,\dots, 10$, this gives different gene subsets ${S}_i$ for 10 cancer studies. Training dataset was employed to find gene subsets specific to each cancer studies. However, it will be difficult to apply a ML technique for computing classification accuracy using different genes belonging to different cancer studies. Since for ML techniques, the same features (or genes) should be employed, it is not possible to find classification accuracy by using class specific features obtained from Equation (3). Therefore, we took a union of all gene subsets $S={\cup}_{i=1}^{10}{S}_i$ and used subset $S$ to find classification accuracy using a RF classifier. The validation set is used to tune the hyperparameters of the RF, and a separate test set is employed to perform evaluation. We used liblinear package in MATLAB to implement L2 regularized logistic regression (https://www.csie.ntu.edu.tw/∼cjlin/liblinear/).

### Experimental setup

The dataset was partitioned into 80:10:10 segments corresponding to training set, validation set and an independent test set, respectively. The training set is employed to achieve model fitting, whereas the validation set is used to evaluate its fitness. This picks the hyperparameters for which the validation error is small. The independent test set is held aside and applied to provide an unprejudiced evaluation of the final model. The DeepFeature model is implement on Matlab R2020a version software. The appropriate links are given under Code Availability section.

### Performance evaluation

The main aim of this experiment is to show that a subset of imperative elements or genes of nonimage data can be selected by CNN with the utilization of DeepFeature method. We have used high-quality reference resources for enrichment analysis like Reactome and Molecular Signatures Database (MSigDB) [63, 64] to discover the importance of the selected genes to phenotypes by the proposed deep-learning based technique. DeepFeature can find different subset of genes for each of the phenotypes.

We compared our methodology with alternative ML techniques. A number of ML algorithms exist to find a subset of genes [65, 66]. We applied ANOVA, Lasso [67], highly variable genes method [68] and logistic regression [3] for feature selection and RF as a classifier. The feature selection step was applied on the training set. This gives a subset of genes. Since enrichment analysis usually requires a smaller set of genes, the aim is to find roughly ~1500 genes. Thereafter, hyperparameters of RF is tuned using training set on a subset of selected genes, and model fitness is evaluated using the validation set. The classification accuracy was from the independent test set.

### Evaluation of feature selection capabilities

As outlined above, both DeepFeature and other exemplar algorithms offer some functionality for reducing the number of genes to a more focused subset enriched for the genes used for correctly identifying the relevant cancer study. This feature selection is of particular importance in biological data analysis where identification of key genes and underlying mechanisms is usually part of the overall goal—especially for tasks like identification of clinically useful sparse diagnostic signatures. To evaluate the utility of DeepFeature from this perspective, we have quantified the enrichment of ‘gold standard’ cancer specific gene sets and pathways from MSigDB (C6 subset). In all instances the enrichment was calculated using Fisher’s exact test and reported *P*-values were corrected for multiple testing using Benjamini–Hochberg FDR method. In addition, we report the housekeeping gene counts based on the annotation from The Human Protein Atlas (THPA, http://www.proteinatlas.org) [69] and a study by Eisenberg and Levanon [70]. Here, the assumption is that a more relevant selection would tend to have fewer housekeeping genes.

### Running the DeepFeature algorithm

Analyzing a large number of elements will cause overlaps in the small pixel frame and it becomes challenging to perform feature selection. This can cause important elements to be overlooked in the selection process. Therefore, it is useful to perform element reduction to reach a manageable size due to the pixel frame size and hardware limitations.

The element arrangement step of DeepFeature utilizes t-SNE (with or without Snowfall) and PHATE. In the case of t-SNE technique, it supports various distance measures, $dis{t}_j$. In this study, $dis{t}_j$ are Chebyshev, cosine, correlation and Hamming. A gene set, $G$, processed to DeepFeature with a distance $dis{t}_j$ of t-SNE, gives a gene subset ${g}_j$. Since four distances are adopted, a union of gene subsets are retrieved, i.e. $\hat{g}={\cup}_{j=1}^4{g}_j$. Furthermore, the gene subset $\hat{g}$ is sent to DeepFeature with Hamming distance until a subset of around 1500 genes are obtained (see Supplementary Figure S1.1 and corresponding discussion in Supplementary File 1). Thereafter, gene set and pathway enrichment analyses were performed to evaluate overall relevance of the recovered gene set with respect to current knowledge.

**Key Points**

- In this paper, we present, DeepFeature, a more advanced version of our previous work, DeepInsight, that can now recover underlying feature combinations specific to each class of interest. DeepInsight (which is part of a winning model in Kaggle.com organized by MIT and Harvard, and applied in many fields of research), established a novel approach that allows high-throughput biological data to be represented in a form compatible with current state-of-art convolutional neural network (CNN) architectures.
- In addition to delivering high classification performance, DeepFeature also offers a powerful means for the identification of biologically-relevant gene sets.
- DeepFeature converts non-image samples of RNA-seq data into image-form, and, furthermore, performs gene selection via CNN. To our knowledge, this is the first approach to employ CNN for element or gene selection on non-image data.
- When applied to the task of cancer classification, our DeepFeature approach was able to identify coherent sets with significant enrichment of genes in cancer-associated pathways from MSigDB and a gold-standard reference set. Further analysis of these results suggested biologically meaningful connections of potential interest to our understanding of the differences between major cancer types.
- DeepFeature is available for download and the web-links provided in the paper.

## Supplementary Material

Supplement_File_1_2_4_bbab297

SupplementFile_3_bbab297

## Code availability

DeepFeature software package (in Matlab), dataset, installation instructions and user-manual are available from the GitHub link https://alok-ai-lab.github.io/deepfeature/. The dataset is also separately available from the link http://emu.src.riken.jp/DeepInsightFS/dataset1.mat, note the size is 1.5GB. The following links are also given for software package http://www.alok-ai-lab.com/tools.php and/or http://emu.src.riken.jp/. The supporting tool, DeepInsight, also has released reference implementation in Matlab (http://www.alok-ai-lab.com/tools.php) and Python as pyDeepInsight https://alok-ai-lab.github.io/DeepInsight/. The description of it can also be accessed by visiting the official page of Matlab at https://www.mathworks.com/company/user_stories/case-studies/riken-develops-a-method-to-apply-cnn-to-non-image-data.html

## Notes

**Alok Sharma** is a Senior Research Scientist at RIKEN, Japan; Adjunct Professor at Griffith University, Australia. His research interests are bioinformatics, AI and proteomics. He has published over 140 scientific papers with h-index 37.

**Artem Lysenko** is a Researcher at the RIKEN Laboratory for Medical Science Mathematics. His research is in the areas of biomedical deep learning, applied data science and biological network analysis.

**Keith A. Boroevich** is a research technician at the RIKEN Laboratory for Medical Science Mathematics.

**Edwin Vans** is a PhD student at the University of the South Pacific and a Lecturer at Fiji National University. His research interests are in bioinformatics and machine learning.

**Tatsuhiko Tsunoda** is a Professor at the School of Science, the University of Tokyo (UT). He is leading laboratories for Medical Science Mathematics at UT, Tokyo Medical and Dental University, and RIKEN.
