# Supplementary material for: DeepFeature: feature selection in nonimage data using convolutional neural network
Source: Brief Bioinform. 2021 Aug 6;22(6):bbab297. doi: 10.1093/bib/bbab297 (PMC8575039; doi:10.1093/bib/bbab297)
Supplement: Supplement_File_1_2_4_bbab297 [file supplement_file_1_2_4_bbab297.docx]

**DeepInsight-FS: Selecting features for non-image data using convolutional neural network**

Alok Sharma^1,2,3,&,^*, Artem Lysenko^1,&,^*, Keith A Boroevich^1^, Edwin Vans^3^, Tatsuhiko Tsunoda^1,4,5,6,^*

^1^Laboratory for Medical Science Mathematics, RIKEN Center for Integrative Medical Sciences, Japan.

^2^Institute for Integrated and Intelligent Systems, Griffith University, Australia.

^3^School of Engineering & Physics, University of the South Pacific, Fiji

^4^ Laboratory for Medical Science Mathematics, Department of Biological Sciences, Graduate School of Science, The University of Tokyo, Tokyo, Japan.

^5^Department of Medical Science Mathematics, Medical Research Institute, Tokyo Medical and Dental University, Tokyo, Japan.

^6^ CREST, JST, Tokyo, Japan

* corresponding authors: [alok.fj@gmail.com](mailto:alok.fj@gmail.com), [artem.lysenko@riken.jp](mailto:artem.lysenko@riken.jp), [tsunoda@bs.s.u-tokyo.ac.jp](mailto:tsunoda@bs.s.u-tokyo.ac.jp)

^&^ Equal contributing first author

Supplementary File 1

Feature selection and tuning of DeepFeature parameters

CNN parameter

The SqueezeNet has been used for CNN implementation. The SqueezeNet configuration has a depth of 18. The hyperparameters are obtained by applying the Bayes optimization technique from a range of values. The parameters considered are learning rate, momentum, and L2regularization. Some other parameters are fixed. The maximum objective evaluation and maximum epochs are set to 50 and 15, respectively, for performing the Bayesian optimization technique. Table S1.1 depicts a summary of the parameters used.

Table S1.1: DeepFeature training parameter options for Bayesian optimization technique

| *Variables* | *Values/range* |
| --- | --- |
| Net | SqueezeNet |
| Training option | sgdm |
| InitialLearningRate | [1e-5 1e-1] |
| Momentum | [0.8 0.95] |
| L2regularization | [1e-10 1e-2] |
| Max Objectives | 20~50 |
| MaxTime | 24x60x60 |
| Execution environment | Single-GPU |
| MaxEpochs | 15 |
| Min batch size | 10 |
| Weight learn rate factor | 10 |
| Bias learn rate factor | 10 |
| Image Size | 227 x 227 |

The range of values are applied during the training session, and the best values were selected, which gave the least validation error.

Two types of norms are introduced in DeepInsight [1]. However, in this work, we applied Norm-2.

Dimensionality reduction technique

In many applications, the dimensionality of feature space (i.e. the number of features) is very large. It is, therefore, necessary to reduce the dimensionality for better generalization capability, reduced computational complexity, and retrieving information.

The two major streams of dimensionality reduction techniques (DRTs) are feature selection and feature extraction. Feature selection methods retain only a few important features and discard others. On the other hand, feature extraction methods, construct a few features from the large set through their linear (or non-linear) combination. The target or output, in this case, is usually the weighted sum of inputs (in some cases including biases).

Though CNN performs feature extraction at its layers, we utilized t-SNE [2] and PHATE [3] for finding locations of elements or genes. For t-SNE, if the number of elements or data dimensionality is above 5000, Burneshut algorithm is applied for faster processing; otherwise, the exact algorithm is used. To acquire a variety of elements, we utilized various distances of t-SNE such as cosine, correlation, Chebyshev and hamming.

According to Moon et al. [3], PHATE generates a low-dimensional embedding specific for visualization, which provides an accurate, denoised representation of both local and global structure of a dataset in the required number of dimensions without imposing any strong assumptions on the structure of the data, and is highly scalable both in memory and runtime. Therefore, we also adopted PHATE as one of DRTs for DeepFeature modelling.

Element versus feature

In this work, the term ‘element’ refers to the raw data, and ‘feature’ refers to the processed or extracted data. However, the term ‘feature’ encompasses ‘element’ since element becomes feature if identify transform is applied, i.e., $feature=identity\times element$.

The term ‘element arrangement’ in this paper, is used because we utilized RNA-seq data. However, this term can be considered identical to ‘feature arrangement’.

Feature mapping

After determining the element locations using the training set, the mapping of elements is carried on to these locations. In a situation where more than one elements occupy the same spot, then their averaged values are utilized; for instance, if locations of $g_{i},g_{j}$ and $g_{k}$ are the same $(r,c)$ then $(g_{i}+g_{j}+g_{k})/3$ will be mapped on this location. In this case, quantized compression will occur. Only the training set is used to find element locations. The validation and test sets used the same locations obtained from the training set. In regards to the empty pixels; i.e., the pixel locations that do not have any elements are referred here as Base, and its value is fixed as 1.

Feature selection using DeepFeature

The RNA-seq data was pre-filtered to reduce to 19086 protein-coding genes. In the training set, several genes have zero expression values, and such 73 genes have been removed. This gives 19013 genes. After that, DeepFeature was used to produce around 1500 genes for enrichment analysis. As discussed under section ‘Running the DeepInsight-FS algorithm’ of the manuscript, DeepFeature runs in multiple stages to acquire the desired number of elements. Figure S1.1 shows an overall framework of DeepFeature with various distances used when t-SNE (with or without Snowfall algorithm) was used. These distances were not applied in the case of PHATE method.

Figure S1.1: An overall framework of DeepFeature execution scheme (when applying t-SNE).

**1) DeepFeature with t-SNE and Snowfall algorithm**

The RNA-seq data, with 19,013 genes, is processed to DeepFeature. When t-SNE is applied with Snowfall algorithm, different distances is used at *step 1*, such as cosine, correlation, Chebyshev and hamming distances. As a result, gene subsets $g_{1}$, $g_{2}$, $g_{3}$ and $g_{4}$ are found. The union of these four subsets is $\hat{g}$, which is then further processed to DeepFeature with hamming distance at *step 2*, and obtain a final subset $\hat{g}_{r}$. The details of hyperparameters selected for each run and the number of genes at *step 1* are listed in Table S1.2, and for *step 2* are listed in Table S1.3. The union of genes, $\hat{g}_{r}$, retrieved from ­*Step 1* is 5228. Since the gene size is much larger than the desired genes (~1500), *step 2* is conducted to reduce the size further. For *step 2*, the input gene size is 5228, and the output is 1806. The classification accuracy on the independent test set with the selected gene subset is 98%.

Table S1.2: Selected hyperparameters and number of genes selected during each stage of DeepFeature in *Step 1* when t-SNE and Snowfall are applied.

Table S1.3: Selected hyperparameters and number of genes selected in *step 2* for DeepFeature (t-SNE+Snowfall).

Figure S1.2 shows an input RNA-seq sample having 5228 genes (left) and its activations at the output (right). The preferential order of activation is from red to yellow to blue; i.e., the genes under the red activation zone is the most desirable. We have only considered genes corresponding to the red activation and discarded the other regions such as yellow, blue and without activation. After processing all the training set, we get 1806 genes in total for all the 10 cancer studies.

DeepFeature gives different gene subsets for different phenotypes. This is depicted in Figure S1.3. In this figure, class 1 to class 10 refer to cancer studies TCGA-BRCA, TCGA-COAD, TCGA-HNSC, TCGA-KIRC, TCGA-LGG, TCGA-LUAD, TCGA-LUSC, TCGA-PRAD, TCGA-THCA, and TCGA-UCEC, respectively. For these cancer studies, the DeepFeature method selects the following number of genes (listed in the same order of cancer studies as mentioned above): 1444, 1147, 1277, 1341, 936, 1135, 1167, 1210, 1036 and 1294, respectively. For many cancer studies, the same or common genes appeared, and the union of genes belonging to 10 cancer studies is 1806.

It can be observed from Figure S1.3 that most of the genes to represent cancers are same. However, some genes are different, which helps in identifying different cancers. Therefore, the DeepFeature method finds genes which are representative and at the same time different for each cancer study.


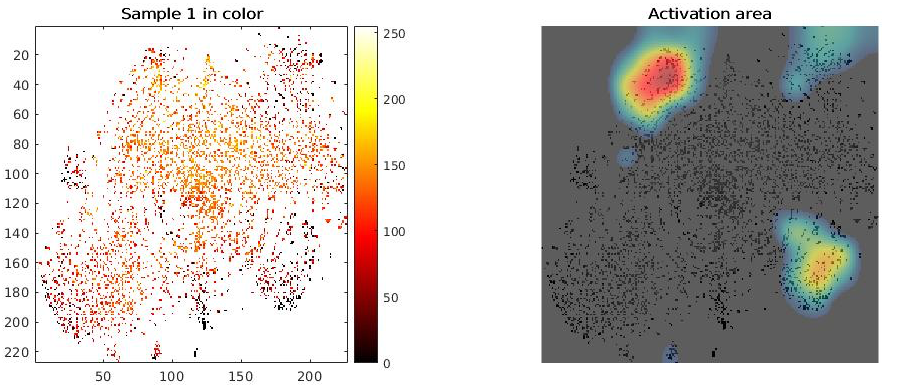


Figure S1.2: DeepFeature (t-SNE+Snowfall): a converted image sample having 5228 genes (left) and its corresponding activations (right).


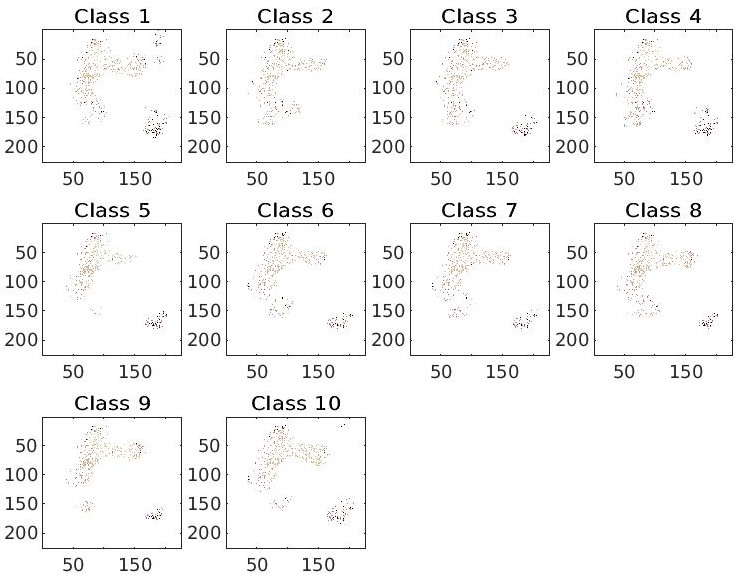


Figure S1.3: Selected gene subsets of each 10 cancer studies of DeepFeature (t-SNE+Snowfall).

**2) DeepFeature with t-SNE** (without Snowfall algorithm used)

As before, DeepFeature with t-SNE (without Snowfall) is also conducted in a similar manner. The detailed results are omitted for brevity. However, summary is given in Table S1.4. Here, we selected two subsets with 1914 genes (Stage5) and 962 genes (Stage6).

Table S1.4: Selected hyperparameters and number of genes selected in *step 2* for DeepFeature (with t-SNE).

Figure S1.4 shows an input RNA-seq sample having 3278 genes (left) and its activations at the output (right). The preferential order of activation is from red to yellow to blue. As before, only the red activation region is considered.

Figure S1.4: DeepFeature (t-SNE without Snowfall): a converted image sample having 3278 genes (left) and its corresponding activations (right).

In a similar manner, a subset of genes for a class is depicted in Figure S1.5. Each cancer study has a subset of genes; i.e., DeepFeature is providing class-dependent features.


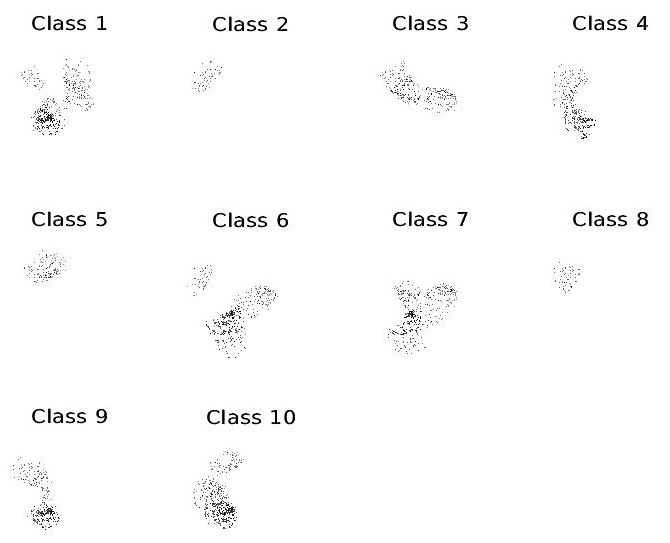


Figure S1.5: Selected gene subsets of each 10 cancer studies of DeepFeature (t-SNE without Snowfall).

**3) DeepFeature with PHATE**

DeepFeature with PHATE is also conducted. For PHATE, step 1 is redundant and results can be found by directly applying Step 2 (ref. Figure S1.1). Summary of results is given in Table S1.5. Here, 1516 genes are selected.

Table S1.5: Selected hyperparameters and number of genes selected for DeepFeature (when using PHATE).

Figure S1.6 shows an input RNA-seq sample having 13873 genes (left) and its activations at the output (right). The preferential order of activation is from red to yellow to blue. As before, only the red activation region is considered.

Figure S1.6: DeepFeature (with PHATE): a converted image sample having 13873 genes (left) and its corresponding activations (right).

A subset of genes for a class is depicted in Figure S1.7. Each cancer study has a subset of genes; i.e., DeepFeature is providing class-dependent features.


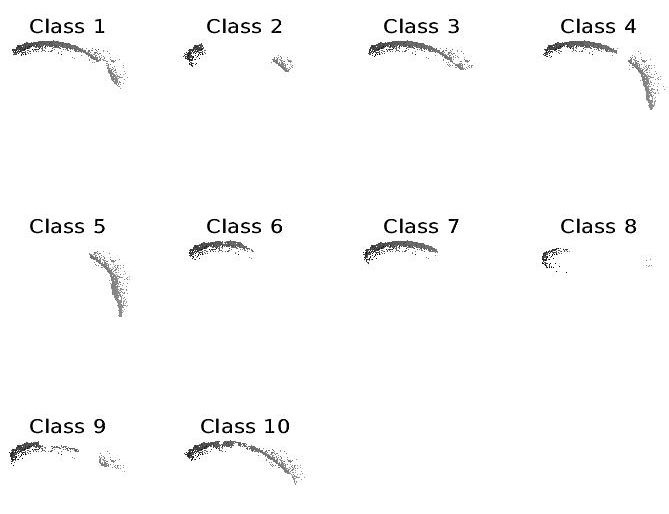


Figure S1.7: Selected gene subsets of each 10 cancer studies of DeepFeature with PHATE.

Supplementary File 2 - Enrichment analysis

Figure S2.1 illustrates gene set overlap by DeepFeature and logistic regression techniques. The gene overlap is highest between the same class. Considerable overlap between adjacent columns of DeepFeature can be seen, depicting similar genes are used to identify different cancer studies.


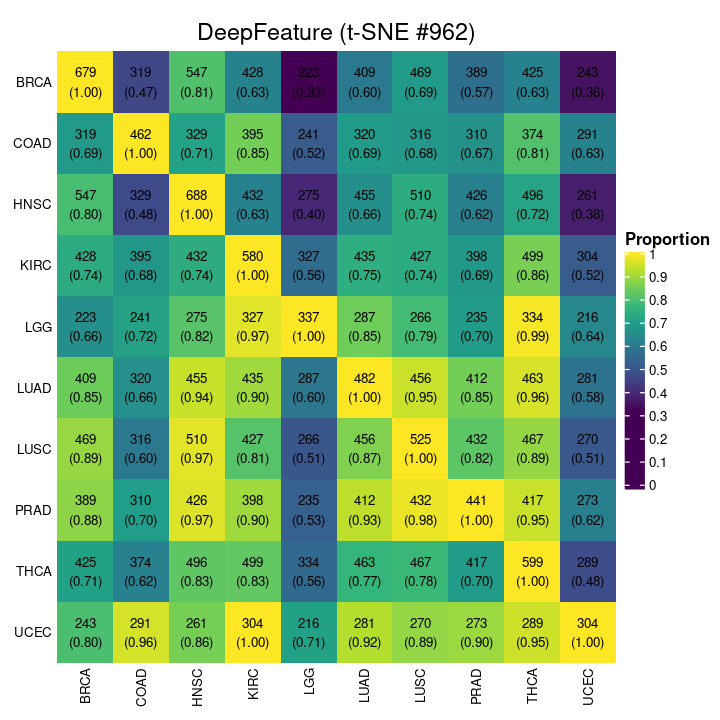

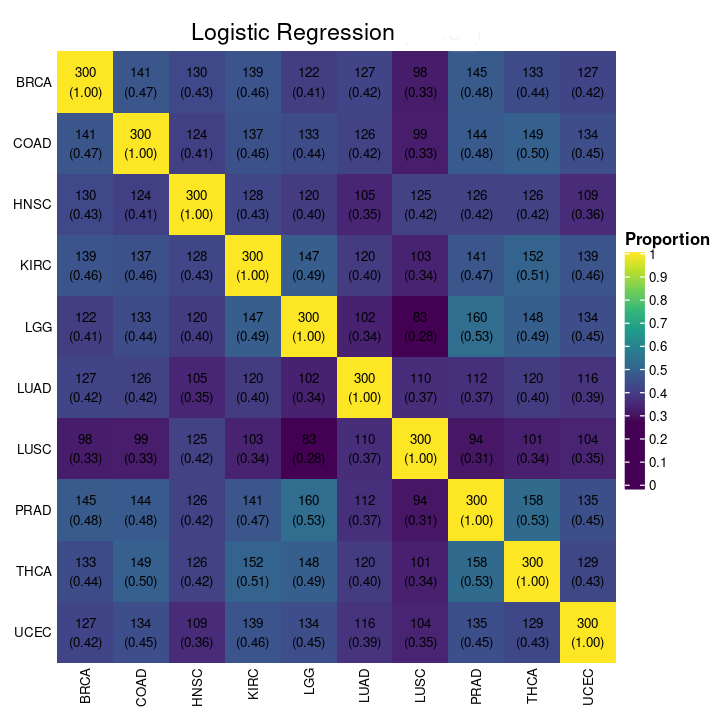


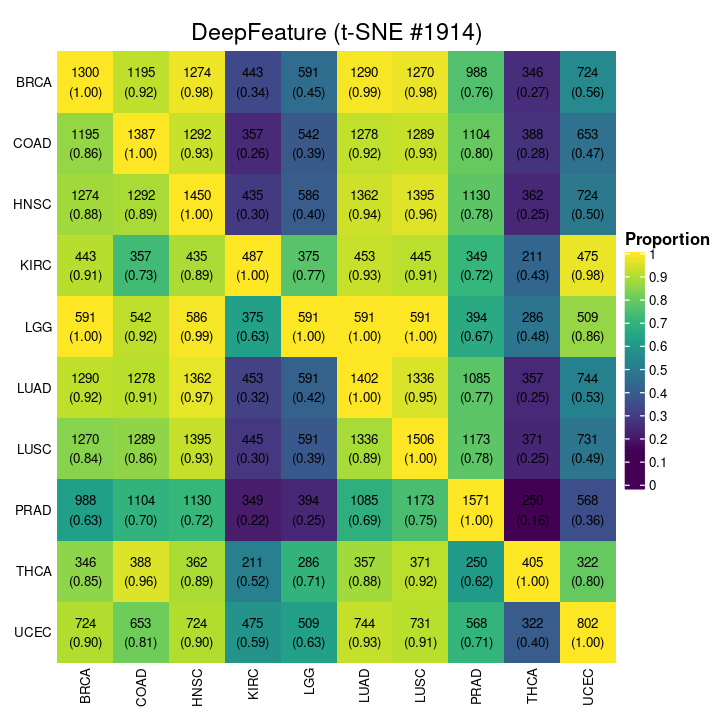

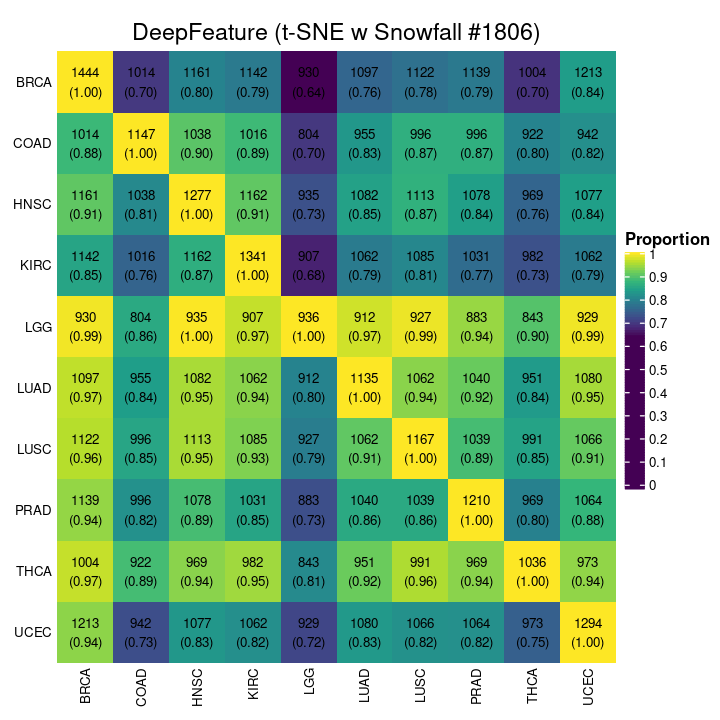


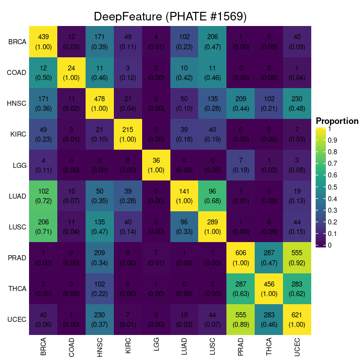


Figure S2.1: Gene set overlap by DeepFeature and logistic regression.

Four types of DeepFeature results are shown following the applications of t-SNE (962 and 1914 gene subsets), t-SNE with Snowfall (1806), and PHATE (1569). It can be observed that gene subsets produced while applying PHATE have the least overlap of genes between different cancer studies.

Figure S2.2 illustrates the performance evaluation of all the methods studied in this work in terms of the number of significant enrichment pathways. We performed MSigDB analysis on the gene sets obtained from different algorithms. It can be seen that ANOVA, Lasso and high-variance genes (HVG) methods did not obtain unique gene sets for individual cancer studies. Nonetheless, logistic regression and DeepFeature produced class-dependent gene subsets. Among DeepFeature, utilizing PHATE produced the least significant enrichment and t-SNE with Snowfall produced the best enrichment pathways.


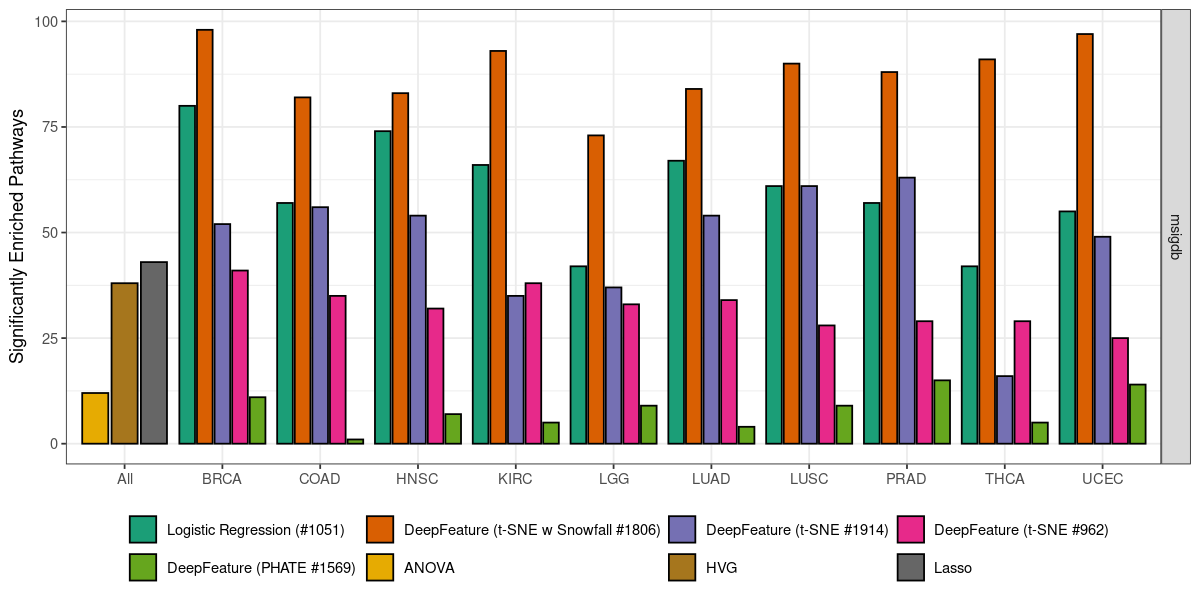


Figure S2.2: MSigDB analysis on ANOVA, Lasso, high-variance genes, logistic regression and DeepFeature. Four strategies are employed to find gene locations for DeepFeature, these are t-SNE (#962 and #1914 genes), t-SNE with Snowfall (#1806), and PHATE (#1569).

Figure S2.3a shows the overlap of gene subsets between several methods studied in this paper. For instance, ANOVA and DeepFeature (with t-SNE+Snowfall) have 143 genes in common, and there are no common genes between ANOVA and HVG. Lasso and DeepFeature (with t-SNE with 1914 genes) have 267 common genes which are the highest among any two methods. Moreover, Figure S2.3b depicts the gene annotation of all the methods. The yellow region shows the selected genes for a particular method (in the column), and the purple region offers genes non-selected. It also indicates HouseKeeping genes. For example, HVG has the least number of HouseKeeping genes compared to other methods tested.


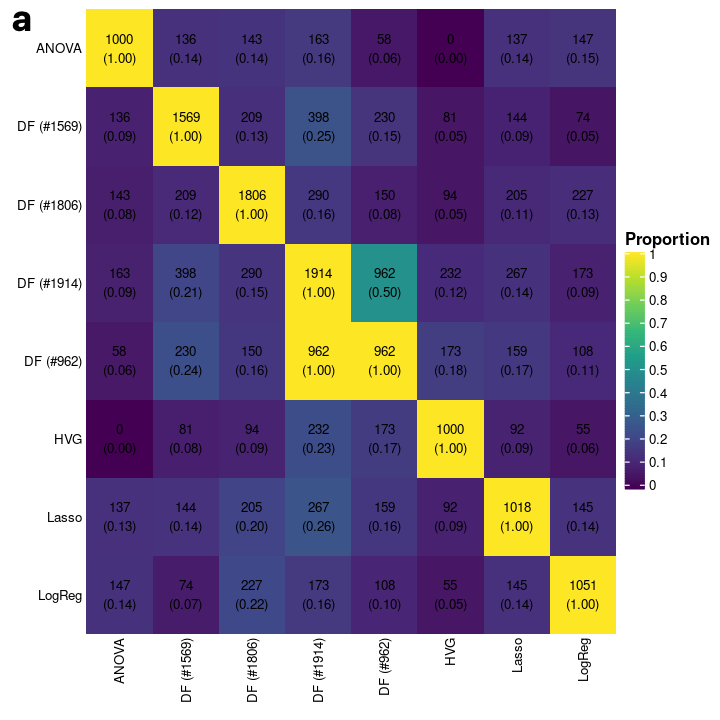

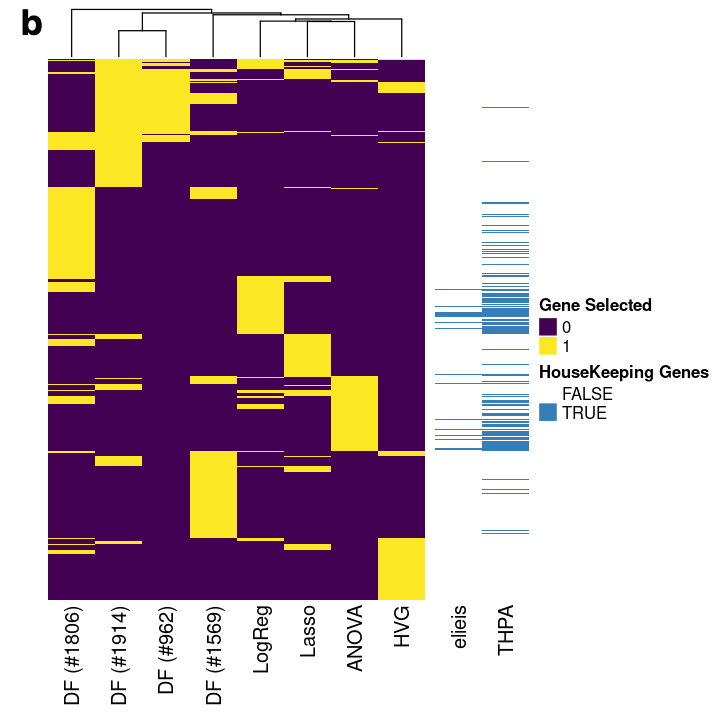


Figure S2.3: a) Overlap of gene subsets between all the methods; and b) gene annotation for all the methods (where DeepFeature with t-SNE+Snowfall is DF(#1806); with t-SNE is DF(#962) and DF(#1914); and with PHATE is DF(#1569).

It can be observed from Figure S2.4 that only marginal overlap occur among gene signatures for all of the cancer studies, indicating that DeepFeature method consistently identified alternative combinations of genes not previously identified in this earlier work.


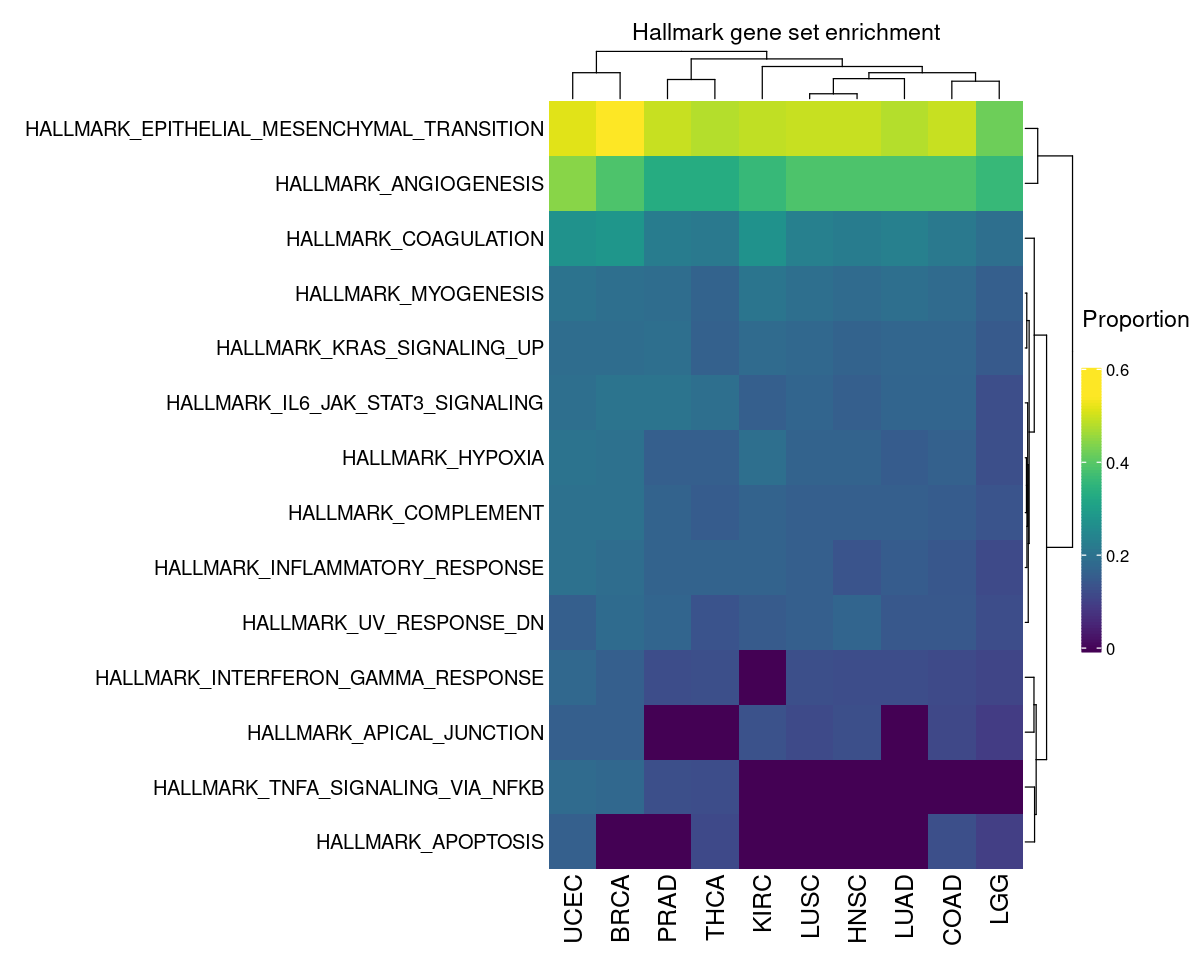


Figure S2.4: Hallmarks of cancer collection from the MSigDB database

Supplementary File 4

Snow-fall compression algorithm

For an illustration, consider Figure S4.1a, where a $6\times6$ pixel frame represents 5 elements or $x\in\mathbb{R}^{5}$. The pixel frame has been divided into 4 quadrants. Suppose the distance of an element $i$ from the center C is depicted as $d_{i}$ then according to Figure S4.1, $x_{3}$, is the closest from the center C (shaded as grey), and $x_{1}$ is the farthest. Since the distance $d_{3}$ is lowest, element $x_{3}$ will first move or fall towards the center, followed by $x_{2}$, $x_{5}$, $x_{4}$, and $x_{1}$. The falling towards the center $C$ will continue until the element reaches $C$ or hit by some other elements on its path. This will free up space and as a result, it is possible to represent more elements in a given pixel frame.

After moving all the elements towards the center, the frame will appear as shown in Figure S4.1b (as blue dots). It can be seen that $x_{3}$ captured the center position, and other elements made free fall to squeeze towards the center of the frame. Now, instead of having a frame size of $6\times6$, we can manage with the frame size of $3\times4$. This frees up more space and new elements can be added (as shown in Figure S4.1b as red dots) or in other words, higher-dimensional data can be considered for the same pixel frame. More elements, e.g. $x_{6},x_{7}$ and $x_{8}$, are added to the same frame in Figure S4.1b after falling of elements towards $C$.

In this algorithm, the elements fall towards the center of the frame, and hence we call it a snow-fall compression algorithm. In the next section, we discuss the mathematics of the snow-fall algorithm.

**Figure S4.1**: a) An illustration of 5 elements $x_{i}$ (for $i=1,2,\ldots,5$) in a 6x6 pixel frame (note rows are depicted in negatives so that cartesian coordinates and pixel coordinates can be represented simultaneously). b) Squeezing all the elements towards the center, and adding new elements or features after compression (red dots).

***Basis of Snow-Fall Algorithm***

Let $U=\{u_{1},u_{2},\ldots,u_{d}\}$ be the vectors in 2D pixel frame. The first step is to find a quadrant where a vector or point $u={(x}_{u},y_{u})\in U$. In Figure S4.2a, the four quadrants are shown and conditions are given for when a point $u$ will come under one of these 4 quadrants. The center point is depicted as $u_{c}=(x_{c},y_{c})$ and the edge point is $u_{p}=(x_{p},y_{p})$.

Given points $u$, $u_{c}$ and $u_{p}$, the quadrant conditions are specified as

1. Q1 if $x_{p}\leq x_{c}$ and $y_{c}\leq y_{p}$, find point $u$ where

$x_{p}\leq x_{u}\leq x_{c}$, and

$y_{c}\leq y_{u}\leq y_{p}$

1. Q2 if $x_{p}\leq x_{c}$ and $y_{p}<y_{c}$, find point $u$ where

$x_{p}\leq x_{u}\leq x_{c}$, and

$y_{p}\leq y_{u}<y_{c}$

1. Q3 if $x_{p}>x_{c}$ and $y_{c}\geq y_{p}$, find point $u$ where

$x_{p}\geq x_{u}>x_{c}$, and

$y_{c}\geq y_{u}>y_{p}$

1. Q4 if $x_{p}>x_{c}$ and $y_{p}>y_{c}$, find point $u$ where

$x_{p}\geq x_{u}>x_{c}$, and

$y_{p}\geq y_{u}>y_{c}$

**Figure S4.2**: a) Quadrant analysis to locate a point $(x_{u},y_{u})$ (note the quadrant numbers are selected as per convenience and it may not be identical to angular quadrants). The frame is adjusted such that no point has negative values. The edge point is depicted by $(x_{p},y_{p})$ and center as $(x_{c},y_{c})$ b) An illustration of points under subarea A, where $u_{r}=(x_{r},y_{r})$ is the closest point to $u_{p}=(x_{p},y_{p})$, and $u_{i}$ is the intersection point between the two perpendicular lines.

In order to develop the model, let us consider quadrant 1 (Q1) as depicted in Figure S4.2b. Let $A$ be the subarea in Q1, $u_{c}$ be the center point, $u_{p}$ be the edge point of $A$, and $u_{r}$ be the closest point to $u_{p}$ given the subarea $A$.

In $A$, equation of a line emanating from $u_{p}$ and passing at the center can be depicted as

$y=mx+(y_{c}-mx_{c})$ (1)

Assuming no points in $A$ exist other than $u_{p}$, then

Case1) No point at $u_{c}=\left( x_{c},y_{c} \right)$ exists and therefore $u_{p}=(x_{p},y_{p})$ will fall at the location of $u_{c}$, i.e.,

$u_{c}\leftarrow u_{p}$.

Case 2) If a point $u_{c}$ exist at the center, then

If $x_{p}<x_{c}$, then

$x_{p}^{'}=x_{c}-0.5$, where $x_{p}^{'}$ is a new location of $x_{p}$, and from Eq (1)

$y_{p}^{'}=[mx_{p}^{'}+\left( y_{c}-mx_{c} \right)]$, where $[\cdot]$ is either *floor* or *ceil* of a numerical value.

If $x_{p}>x_{c}$, then

$x_{p}^{'}=x_{c}+0.5$, and from Eq (1)

$y_{p}^{'}=[mx_{p}^{'}+\left( y_{c}-mx_{c} \right)]$

If other points also exist as depicted in Figure S4.2b, then the model will search the closest distance from $u_{p}$ to all $u\in A$, i.e.,

$r=\arg\min_{u_{j}\in A} (u_{p},u_{j})$ (2)

This will return point $u_{r}$, a point closest to $u_{p}$.

Now a perpendicular line to Eq (1) and passing $u_{r}$ can be given as

$y=-\frac{1}{m}x+(y_{r}+\frac{1}{m}x_{r})$ (3)

The intersection point $(x_{i},y_{i})$ of Eq (1) and Eq (3) can be found by substituting Eq (3) in Eq (1) (see the intersecting point as a black dot in Figure S4.2b), we get

$x_{i}=\frac{\left( y_{r}+\frac{1}{m}x_{r} \right)-\left( y_{c}-mx_{c} \right)}{m+\frac{1}{m}}$ (4)

Substituting $x_{i}$ in Eq (1), we get

$y_{i}=\frac{m\left( \left( y_{r}+\frac{1}{m}x_{r} \right)-\left( y_{c}-mx_{c} \right) \right)}{m+\frac{1}{m}}$ (5)

Therefore, in this case, the new location for $u_{p}=(x_{p},y_{p})$ would be $u_{p}^{'}=u_{i}=(x_{i},y_{i})$.

Note that these $x_{i}$ and $y_{i}$ are in Cartesian coordinates, and it can move to at most 4 locations in pixel coordinates (shown by the arrow in Figure S4.2b).

One way of finding the location for intersection point in the pixel coordinate is to compute distance $d_{pi}$ between $u_{p}$ and $u_{i}$ as

$d_{pi}=\sqrt{\left( x_{p}-\left[ x_{i} \right] \right)^{2}+\left( y_{p}-\left[ y_{i} \right] \right)^{2}}\leq d_{pr}$ (6)

Where $[\cdot]$ is either *floor* or *ceil*, and $d_{pr}$ is the distance between $u_{p}$ and $u_{r}$; i.e.,

$[x_{i}]$ $=$ $floor(x_{i})$ $floor(x_{i})$ $ceil(x_{i})$ $ceil(x_{i})$

$[y_{i}]$ $=$ $floor(y_{i})$ $ceil(y_{i})$ $floor(y_{i})$ $ceil(y_{i})$ (7)

(distance) $d_{ff}$ $d_{fc}$ $d_{cf}$ $d_{cc}$

This gives 4 conditions, and the best condition would be $d_{pi}<d_{pr}$. Suppose $d_{ff}<d_{fc}<d_{cf}<d_{pr}<d_{cc}$, then select condition $cf$ and accordingly select the new location of $u_{p}$; i.e., $u_{p}^{'}=(ceil\left( x_{i} \right),floor\left( y_{i} \right))$.

Another way of finding the location can be considered by using the center as a reference point. Let $d_{ci}$ be the distance between the center and the intersection point, we have

$d_{ci}=\sqrt{\left( x_{c}-\left[ x_{i} \right] \right)^{2}+\left( y_{c}-\left[ y_{i} \right]^{2} \right)}$ (8)

In a similar way as Eq (7), $[\cdot]$ in Eq (8) can be either *floor* or *ceil*, giving 4 conditions. The nearest point towards the center would be

$\left( c^{'},i^{'} \right)=\arg\min_{ff,fc,cf,cc} d_{ci}$ (9)

These steps have to be taken for other points, making this algorithm an iterative one until all the points fall.

***Intersecting points when*** $\boldsymbol{m=0}$ ***and*** $\boldsymbol{m=\infty}$

Next, we see the intersecting point (Eq (4) and Eq (5)) in extreme condition; i.e., when $m=\infty$ and $m=0$.

if $m=\infty$ then from Eq (4)

$x_{i}=\frac{\left( y_{r}+\frac{1}{m}x_{r} \right)-\left( y_{c}-mx_{c} \right)}{m+\frac{1}{m}}$ $=\frac{\infty}{\infty}$ (intermediate terms)

$x_{i}=\lim_{m\to\infty} \frac{y_{r}}{m+\frac{1}{m}}+\frac{x_{r}}{m^{2}+1}-\frac{y_{c}}{m+\frac{1}{m}}+\frac{x_{c}}{1+\frac{1}{m^{2}}}$

$x_{i}=x_{c}$

Similarly, from Eq (5)

$y_{i}=\frac{m\left( \left( y_{r}+\frac{1}{m}x_{r} \right)-\left( y_{c}-mx_{c} \right) \right)}{m+\frac{1}{m}}+\left( y_{c}-mx_{c} \right)=\frac{\infty}{\infty}$

$y_{i}=\lim_{m\to\infty} \frac{y_{r}}{1+\frac{1}{m^{2}}}+\frac{x_{r}}{m+\frac{1}{m}}+\frac{y_{c}}{m^{2}+1}-\frac{mx_{c}}{m^{2}+1}$

$y_{i}=y_{r}+0+0-\lim_{m\to\infty} \frac{x_{c}}{2m}$ *(from L’Hopital’s Rule)*

$y_{i}=y_{r}$

If $m=0$ then $x_{i}=x_{r}$ and $y_{i}=y_{c}$.

***Non-quantized and Quantized Compression***

The snow-fall algorithm gives an optimum pixel frame of size $p\times q$. The compression would be non-quantized if the desired frame size $m\times n$ is greater than $p\times q$ ($p\leq m$ and $q\leq n$). The compression can be quantized if $p>m$ and $q>n$. The quantized compression can be attained by adjusting the horizontal and vertical sizes of the pixel frame. In this case, it is possible that more than 2 elements can attain one location giving the average value of elements at this particular location. For an instance, if *k* elements $\{g_{1},g_{2},\ldots,g_{k}\}$ having a common location then average value $\frac{1}{k}\sum_{i=1}^{k} g_{i}$ will be mapped at this location.

An illustration of a pixel-frame of size $100\times100$ extracted by DeepInsight is shown in Figure S4.3a. Its non-quantized form by snow-fall algorithm of size $15\times14$ is depicted in Figure S4.3b, and its quantized compression form of size $8\times7$ is given in Figure S4.3c. A video illustration (Video 1) can be accessed as *SnowFall_Video.avi* (note the speed in the video has been reduced for illustration purpose). In the case of quantized compression, multiple elements or genes occupy a common location.

**Figure S4.3**: An illustration of the snow-fall algorithm a) $100\times100$ pixel frame, b) non-quantized snow-fall compression, and c) quantized snow-fall compression.


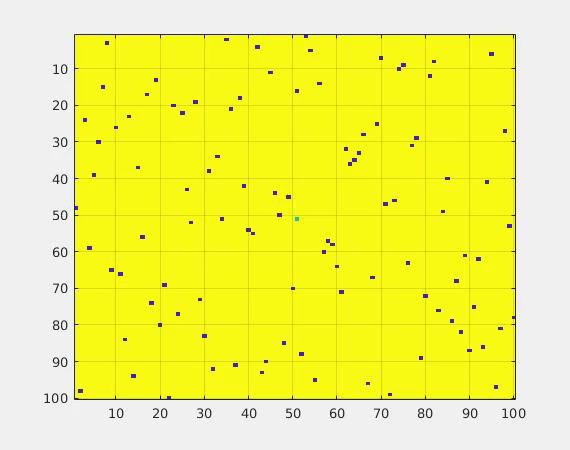


Video 1: A run of the snow-fall compression algorithm.

**Reference**

[1] A. Sharma, E. Vans, D. Shigemizu, K. A. Boroevich, and T. Tsunoda, "DeepInsight: A methodology to transform a non-image data to an image for convolution neural network architecture," *Scientific Reports,* vol. 9, no. 1, 2019, doi: 10.1038/s41598-019-47765-6.

[2] L. v. d. Maaten and G. Hinton, "Visualizing data using t-SNE," *Journal of Machine Learning Research,* vol. 9, no. Nov, pp. 2579-2605, 2008.

[3] K. R. Moon *et al.*, "Visualizing structure and transitions in high-dimensional biological data," *Nat Biotechnol,* vol. 37, no. 12, pp. 1482-1492, 2019, doi: 10.1038/s41587-019-0336-3.
